# Protein pyrophosphorylation by inositol phosphates: a novel post-translational modification in plants?

**DOI:** 10.3389/fpls.2024.1347922

**Published:** 2024-02-22

**Authors:** Yeshambel Emewodih Mihiret, Gabriel Schaaf, Marília Kamleitner

**Affiliations:** Department of Plant Nutrition, Institute of Crop Science and Resource Conservation, Rheinische Friedrich-Wilhelms-Universität Bonn, Bonn, Germany

**Keywords:** inositol pyrophosphates, serine phosphorylation, protein kinase CK2, pyrophosphoproteomics, non-canonical phosphorylation

## Abstract

Inositol pyrophosphates (PP-InsPs) are energy-rich molecules harboring one or more diphosphate moieties. PP-InsPs are found in all eukaryotes evaluated and their functional versatility is reflected in the various cellular events in which they take part. These include, among others, insulin signaling and intracellular trafficking in mammals, as well as innate immunity and hormone and phosphate signaling in plants. The molecular mechanisms by which PP-InsPs exert such functions are proposed to rely on the allosteric regulation via direct binding to proteins, by competing with other ligands, or by protein pyrophosphorylation. The latter is the focus of this review, where we outline a historical perspective surrounding the first findings, almost 20 years ago, that certain proteins can be phosphorylated by PP-InsPs *in vitro*. Strikingly, *in vitro* phosphorylation occurs by an apparent enzyme-independent but Mg^2+^-dependent transfer of the β-phosphoryl group of an inositol pyrophosphate to an already phosphorylated serine residue at Glu/Asp-rich protein regions. Ribosome biogenesis, vesicle trafficking and transcription are among the cellular events suggested to be modulated by protein pyrophosphorylation in yeast and mammals. Here we discuss the latest efforts in identifying targets of protein pyrophosphorylation, pointing out the methodological challenges that have hindered the full understanding of this unique post-translational modification, and focusing on the latest advances in mass spectrometry that finally provided convincing evidence that PP-InsP-mediated pyrophosphorylation also occurs *in vivo*. We also speculate about the relevance of this post-translational modification in plants in a discussion centered around the protein kinase CK2, whose activity is critical for pyrophosphorylation of animal and yeast proteins. This enzyme is widely present in plant species and several of its functions overlap with those of PP-InsPs. Until now, there is virtually no data on pyrophosphorylation of plant proteins, which is an exciting field that remains to be explored.

## 1 Introduction to inositol pyrophosphates: definition, metabolism and functions

**Figure 1:**
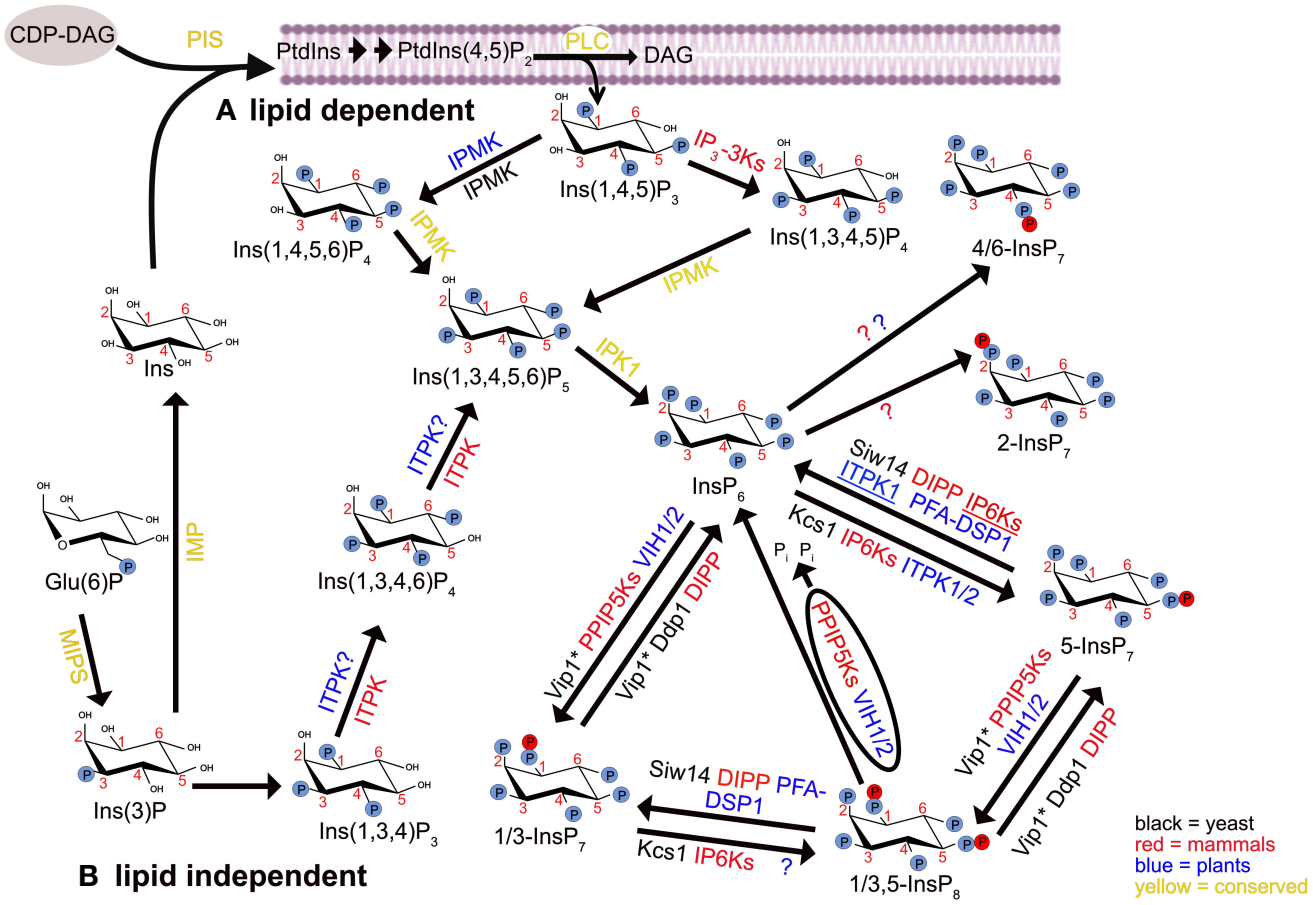
The synthesis and metabolism pathways of *myo*-inositol in mammals, plants and budding yeast *Saccharomyces cerevisiae*. Glucose-6-phosphate Glu(6)P is converted to inositol-3-phosphate (Ins(3)P) through the action of *myo*-inositol phosphate synthase (MIPS). Subsequently, Ins(3)P undergoes dual pathways of metabolism. **(A)** Lipid-dependent pathway: In this route, *myo*-inositol (Ins) is generated from Ins(3)P by inositol monophosphatase (IMP). Ins then combines with CDP-diacylglycerol (CDP-DAG) to initiate the *de novo* synthesis of phosphatidylinositol (PtdIns) by phosphatidylinositol synthase (PIS). PtdIns is a precursor for various phosphoinositides (PIPs), inositol phosphates (InsPs), and pyrophosphorylated inositol phosphates (PP-InsPs). **(B)** Lipid-independent pathway: Ins(3)P is converted into different InsPs and PP-InsPs. Enzymes shown in yellow have been proposed to have conserved functions across mammals, plants and yeast for the synthesis of indicated InsPs. Enzymes involved in InsPs and PP-InsPs synthesis and degradation are differentiated by color: black for yeast, red for mammals and blue for plants. The underlined IP6Ks and ITPK1 enzymes possess ADP phosphotransferase activity in mammals and plants, respectively. The function of several enzymes, in particular those involved in lower InsP biosynthesis, is based on *in vitro* activity. The asterisk (*) on Vip1 shows this enzyme has a dedicated kinase and/or phosphatase activity at carbon D-1 position of InsP_7_ and InsP_8_. “P” symbolizes the phosphate group PO_4_^3-^ (or protonated states). The membrane structure is adapted from the “membrane phospholipid bilayer” template, by BioRender.com (2023). Retrieved from https://app.biorender.com/biorender-templates. Arabidopsis has two IPMK isoforms that are named IPK2α and IPK2β respectively.

Inositol phosphates (InsPs) comprise soluble molecules derived from sequential phosphorylation of *myo*-inositol. These molecules gained focus 40 years ago with the discovery that inositol 1,4,5-trisphosphate (Ins[1,4,5]P_3_ or short “IP3”), derived from the phospholipase C-dependent hydrolysis of phosphatidylinositol 4,5-bisphosphate (PtdIns[4,5]P_2_, **Figure 1**), triggers mobilization of intracellular calcium stores in rat pancreatic acinar cells (Streb et al., 1983; Berridge, 1986). Besides its canonical role as a second messenger regulating a plethora of cellular activities in animal cells (Berridge, 1993), more important for the scope of this review is the fact that “IP3” is one of several InsP_3_ isomers (Barker et al., 1995; Brearley and Hanke, 2000) and serves as a precursor of additional InsPs, such as various InsP_4_ and InsP_5_ isomers, as well as InsP_6_ and higher phosphorylated InsP species (Shears, 2018; Shears and Wang, 2020).

InsP_6_ is prone to further phosphorylation, generating a subset of InsPs harboring one or two diphosphate moieties in addition to the monophosphates. These molecules are collectively called “inositol pyrophosphates” (PP-InsPs) and include diphosphoinositol pentakisphosphate (PP-InsP_5_, also simply termed InsP_7_), as well as bis-diphosphoinositol tetrakisphoshate ([PP]_2_-InsP_4_ or InsP_8_) (Shears, 2009; Ganguli et al., 2020). The pathways for PP-InsP biosynthesis rely on two classes of enzymes: i) inositol hexakisphosphate kinases (IP6Ks), which introduce pyrophosphate moieties at position 5 of the inositol ring, and ii) diphosphoinositol-pentakisphosphate kinases (PPIP5Ks), that act on position 1 or 3 (**Figure 1**). That is, InsP_6_ is converted to 5-InsP_7_ by IP6Ks and to 1/3-InsP_7_ by PPIP5Ks, and both enzymes can act on each other’s products to generate 1/3,5-InsP_8_ (Saiardi et al., 2001; Mulugu et al., 2007; Draškovič et al., 2008; Chakraborty, 2018; Lorenzo-Orts et al., 2020; Shears and Wang, 2020). With the exception of the budding yeast (*Saccharomyces cerevisiae*) PPIP5K Vip1 and the human PPIP5K2, which specifically generate 1,5-InsP_8_ (Wang et al., 2012; Capolicchio et al., 2014; Dollins et al., 2020), it is still not clear whether other PPIP5K isoforms in mammals or plants catalyze formation of the phosphoanhydride bond at the 1 or the 3 position. Except in budding yeast, the enantiomer identity of 1/3-InsP_7_ and 1/3,5-InsP_8_ therefore remains unresolved in eukaryotes.

The synthetic pathways are only partially conserved among metazoan, fungi and plants. While humans, for instance, harbor three IP6K isoforms and the budding yeast have a single IP6K gene (*Kcs1*) (Saiardi et al., 1999; Chakraborty, 2018), no sequence-related IP6K homolog exists in higher plants. The lack of a canonical IP6K has kept the origin of plant InsP_7_ unresolved until recently, when two Arabidopsis inositol (1,3,4) trisphosphate 5/6-kinases (ITPKs), namely ITPK1 and ITPK2, were shown to harbor InsP_6_ kinase activity that catalyzes the synthesis of 5-InsP_7_ *in vitro* and *in planta* (Laha et al., 2019; Whitfield et al., 2020; Riemer et al., 2021). Mammalian IP6Ks and the Arabidopsis ITPK1 also possess an ADP phosphotransferase activity (**Figure 1**) that allows specific PP-InsP dephosphorylation upon reduced ATP/ADP ratios, a feature that likely contributes to a more energy-conserving PP-InsP turnover than via dedicated phosphatases, but might also be a way for localized ATP production (Voglmaier et al., 1996; Whitfield et al., 2020; Riemer et al., 2021, 2022).

A dual activity is also present in PPIP5Ks, but in this case the interconversion between substrates and products is mediated by two distinct modules: an N-terminal ATP-grasp kinase domain and a C-terminal phosphatase domain (**Figure 1**). The bifunctionality of these enzymes has first been suggested when this class of enzymes was identified in *S. cerevisiae* (Mulugu et al., 2007). While PP-InsP synthase activity of the N-terminal ATP-grasp kinase domain has been shown in different organisms (Choi et al., 2007; Fridy et al., 2007; Mulugu et al., 2007; Pöhlmann and Fleig, 2010; Desai et al., 2014; Laha et al., 2015; Zhu et al., 2019; Dollins et al., 2020), the role of the C-terminal phosphatase-like domain has remained elusive until work on the fission yeast (*Schizosaccharomyces pombe*) PPIP5K Asp1 corroborated the idea that this domain harbors PP-InsP phosphatase activity (Pöhlmann et al., 2014). PP-InsP 1-phosphatase activity of the C-terminal domain of PPIP5Ks was then more directly demonstrated for Asp1 (Wang et al., 2015) and for the plant VIH1/2 proteins (Zhu et al., 2019).

In addition to the phosphotransferase activity of IP6Ks/ITPK1 and the phosphatase domain of PPIP5Ks, PP-InsP catabolism can also be mediated by stand-alone phosphohydrolases. These include members of the NUDIX hydrolase family named diphosphoinositol polyphosphate phosphohydrolases (DIPPs), and of the dual-specificity phosphatase (DSP) family (**Figure 1**). The first is represented by 5 human isoforms (DIPP1, 2α, 2β, 3α and 3β) that preferentially target the β-phosphate at the 1/3-position (Kilari et al., 2013; Chakraborty, 2018), and by the budding yeast only ortholog DDP1 (Cartwright and McLennan, 1999; Safrany et al., 1999). In addition to PP-InsPs, other substrates, such as polyphosphates and diadenosine polyphosphates, can also be hydrolyzed with different levels of preference by DDP1 (Lonetti et al., 2011; Márquez-Moñino et al., 2021). Also Siw14, a member of the plant and fungi atypical dual-specificity phosphatases (PFA-DSPs) (Romá-Mateo et al., 2007), is a PP-InsP phosphatase in budding yeast, specifically cleaving 5-InsP_7_ at the 5-position (Steidle et al., 2016; Wang et al., 2018). All of the five Arabidopsis PFA-DSPs (AtPFA-DSP1-5) were shown to harbor *in vitro* PP-InsP phosphohydrolase activity that was highly specific towards the 5-β-phosphate (Gaugler et al., 2022; Wang et al., 2022b). Increased AtPFA-DSP1 expression in an Arabidopsis T-DNA activator line and heterologous expression of AtPFA-DSP1 in *Nicotiana benthamiana* both led to a decrease in 5-InsP_7_ and 1,5-InsP_8_ levels, suggesting a DSP-dependent degradation of these PP-InsP species *in planta* as well (Gaugler et al., 2022).

The roles these enzymes and their products play in different biological systems were determined at some extent, unveiling a large versatility of PP-InsPs. In mammals, for instance, these molecules were shown to be involved in several cellular processes, including vesicle trafficking (Saiardi et al., 2002; Illies et al., 2007), energy metabolism (Szijgyarto et al., 2011; Shears, 2018), cell migration (Jadav et al., 2016; Fu et al., 2017) and apoptosis (Koldobskiy et al., 2010; Rao et al., 2014). Numerous pathologies have been associated with deranged activities of PP-InsP-metabolizing enzymes, such as male sterility (Malla and Bhandari, 2017; Fu et al., 2018; Bhat et al., 2024), neurological diseases (Fu et al., 2015), type-2 diabetes and obesity (Chakraborty et al., 2010), sight and hearing loss (Yousaf et al., 2018; Khaled et al., 2019), and different types of cancer (Jadav et al., 2016; Chakraborty, 2018; Lee et al., 2020). In some cases, such as male sterility and neurological diseases, it remains unresolved whether pathologic disorders are caused by deranged PP-InsP metabolism or defective enzyme-independent protein-protein interactions in which these kinases participate.

In yeast, PP-InsPs play key roles in the nucleus, where they were shown to regulate telomere length maintenance (Saiardi et al., 2005), mRNA export (York et al., 1999; Tsui and York, 2010), transcription (Lee et al., 2007; Alcázar-Román and Wente, 2008; Steidle et al., 2020) and kinetochore architecture and entry into mitosis (Kuenzel et al., 2022). Several studies employing yeast mutants with altered PP-InsP metabolism propose that such a regulation is particularly relevant during responses to environmental stress (Morrissette and Rolfes, 2020). For instance, the 5-InsP_7_-deficient mutant *kcs1Δ* is sensitive to osmotic and cell wall stresses (Dubois et al., 2002), and is defective in sporulation and pseudohyphal growth upon carbon and nitrogen starvation (Kloimwieder and Winston, 2011; Norman et al., 2018). On the other hand, the *siw14Δ* yeast strain, whose lack of PP-InsP hydrolase activity results in elevated 5-InsP_7_ and InsP_8_ levels, displays increased resistance to heat, osmotic, oxidative and nutritional stresses (Morrissette and Rolfes, 2020; Steidle et al., 2020).

Also in plants, PP-InsPs are involved in responses to environmental conditions. Recent efforts from several research groups have, for instance, identified InsP_8_ and to some extent 5-InsP_7_ as proxies for inorganic phosphate (Pi) sensing in Arabidopsis, via a mechanism that involves PP-InsP binding to SPX domain-containing proteins (Wild et al., 2016; Dong et al., 2019; Zhu et al., 2019; Land et al., 2021; Riemer et al., 2021; Gulabani et al., 2022). Besides acting as modulators of Pi-homeostasis, PP-InsPs also compose the signaling machinery employed in jasmonic acid (JA)-, salicylic acid (SA)- and auxin-dependent responses, therefore playing important roles in the resistance against pathogens and herbivores (Laha et al., 2015; Gulabani et al., 2022), as well as in plant development (Laha et al., 2022). Interestingly, plant InsP_7_ is dominated by an unusual 4/6-InsP_7_ isomer of unknown function while 1/3-InsP_7_ and 5-InsP_7_, isomers also found in all other eukaryotes, are less abundant (Riemer et al., 2021). In contrast to 5-InsP_7_ and InsP_8_, the 4/6-InsP_7_ isomer is not mis-regulated in a Pi over-accumulating Arabidopsis *pho2* mutant, it does not show the strong overshoot reaction observed for 1/3-InsP_7_ and InsP_8_ after Pi resupply, and is therefore likely not involved in Pi signaling (Riemer et al., 2021). In a recent study, members of the inositol polyphosphate multikinase (IPMK) family have been shown to be involved in 4/6-InsP_7_ synthesis in Arabidopsis and in the liverwort *Marchantia polymorpha* (Yadav et al., 2023). However, since knockouts of the *M. polymorpha* single *IPMK* gene display only an approximately 30% reduction in 4/6-InsP_7_, and because both Arabidopsis and *M. polymorpha ipmk* mutant plants display additional defects in lower InsPs, it remains unresolved whether IPMKs have a direct or rather more indirect role in 4/6-InsP_7_ synthesis.

The type of interaction PP-InsPs engage with proteins determines the mode by which these molecules regulate cellular signaling. PP-InsPs can bind allosterically to proteins or act as competitor ligands to modulate, e.g., their function, localization, stability or subsequent protein interactions (Lee et al., 2020). Several of the previously mentioned SPX domains, for instance, were shown to bind preferentially to 5-InsP_7_ and 1,5-InsP_8_ via conserved basic surface clusters (Wild et al., 2016; Dong et al., 2019; Ried et al., 2021), an association that triggers various responses in different organisms (Wild et al., 2016). In Arabidopsis, for instance, InsP_8_ binding to SPX receptors promotes association of the PP-InsP/SPX complex with MYB-like PHR transcription factors (Wild et al., 2016; Dong et al., 2019; Zhu et al., 2019), known master regulators of phosphate starvation responses (PSRs) (Bustos et al., 2010). These components (i.e., PP-InsP, SPX and PHR) were shown to interact upon Pi-sufficient conditions, thus downregulating the expression of Pi starvation-induced (PSI) genes. In agreement with this, PHR1 dissociates from SPX1 under conditions of low Pi availability, where InsP_8_ levels drop substantially, leading to subsequent dissociation of the PHR transcription factor, which is then able to bind promoters of PSI genes to activate PSRs (Wild et al., 2016; Dong et al., 2019; Zhu et al., 2019; Land et al., 2021; Riemer et al., 2021; Gulabani et al., 2022). Another mode of action exerted by PP-InsPs involves competitive binding against other ligands. For instance, the PtdIns(3,4,5)P_3_-dependent plasma membrane localization of the mammalian serine/threonine kinase Akt takes place via the protein’s pleckstrin homology (PH) domain, and 5-InsP_7_ was shown to weaken this interaction by outcompeting the phosphoinositide ligand (Chakraborty et al., 2010).

PP-InsPs also operate via protein pyrophosphorylation, a non-enzymatic post-translational modification in which pre-phosphorylated serine residues accept the high-energy β-phosphate from PP-InsPs (Ganguli et al., 2020). This process has been reported to regulate several yeast and mammalian proteins, however, until the onset of this review, no studies investigating PP-InsP-dependent pyrophosphorylation of plant proteins have been published. Here we provide an overview on protein pyrophosphorylation by PP-InsPs, including the early discoveries that paved the way for this exciting research field. We also discuss the reasoning behind the skepticism in coining protein pyrophosphorylation as a novel post-translational modification, and describe how recent advances in mass-spectrometry helped to finally identify pyrophosphorylated proteins from complex samples. At last, we provide insights into physiological processes in plants that may potentially be regulated by PP-InsP-mediated protein pyrophosphorylation, with a focus on the protein kinase CK2.

## 2 PP-InsPs as protagonists of a novel post-translational modification

The first time a possible involvement of PP-InsPs in phosphotransfer reactions was proposed dates over 30 years ago (Stephens et al., 1993). However, it was only almost a decade later that a PP-InsP species, namely InsP_7_, was shown to in fact phosphorylate eukaryotic proteins *in vitro* (Saiardi et al., 2004). By incubating radiolabeled PP-InsP (i.e., 5β[^32^P]InsP_7_) with extracts of mouse brain and budding yeast, the authors detected various phosphorylated proteins, some of which were isolated and identified by mass spectrometry. Three InsP_7_ targets in budding yeast, i.e., the nucleolar proteins NSR1 and SRP40, and the uncharacterized protein YGR130c, were then further evaluated, leading to important conclusions that laid the ground for subsequent studies on protein pyrophosphorylation by PP-InsPs. These include determining some of the hallmarks of this process: i) the phosphotransfer occurs non-enzymatically, ii) it depends on divalent cations with a preference for Mg^2+^, and iii) protein targets should harbor serine-rich domains flanked by acidic amino acids (Saiardi et al., 2004).

In a follow-up study, the Snyder group also observed that the nucleolar proteins isolated in the Saiardi et al. (2004) work were not phosphorylated by 5β[^32^P]InsP_7_ when ectopically expressed in *E. coli*, in contrast to the robust phosphorylation detected in yeast-purified proteins (Bhandari et al., 2007). This raised the question of which factor is exclusively found in the eukaryotic system that allowed proteins to be phosphorylated by InsP_7_. The authors differentially treated a bacteria-expressed NSR1 fragment with yeast extracts and/or with the serine/threonine protein kinase CK2 (previously called casein kinase II), and confirmed that InsP_7_ can only phosphorylate targets that had been previously undergone CK2-dependent phosphorylation (Bhandari et al., 2007). By employing *in vitro* phosphorylation assays of synthetic peptides harboring either methylated or pyrophosphorylated serines, combined with mass spectrometry analyses, important conclusions could then be drawn. First, InsP_7_ exclusively modifies serine residues via pyrophosphorylation. Moreover, the properties of the InsP_7_-derived pyrophosphate bonds differ from those in phosphoserines in terms of acid lability and phosphatase resistance. While pyrophosphoserines are more easily hydrolyzed by HCl than ATP-derived phosphoserines, they remained inert to enzymatic phosphatase treatment (Bhandari et al., 2007). The authors also tested whether PP-InsPs other than 5-InsP_7_ could pyrophosphorylate proteins. For that, radiolabeled products of the budding yeast PPIP5K Vip1, at the onset of that work claimed to be 4/6-InsP_7_ and InsP_8_, were used. Both PP-InsPs, later found to correspond to the 1-InsP_7_ and 1,5-InsP_8_ isomers (Lin et al., 2009; Shears et al., 2017; Dollins et al., 2020), could indeed pyrophosphorylate proteins *in vitro* indistinctively from 5-InsP_7_ (Bhandari et al., 2007). Such findings suggest a general role of PP-InsPs in regulating protein function via pyrophosphorylation.

## 3 Functional relevance of protein pyrophosphorylation by PP-InsPs

Only a handful of studies address the functional consequences of protein pyrophosphorylation by inositol pyrophosphates (Azevedo et al., 2009; Szijgyarto et al., 2011; Thota et al., 2015; Chanduri et al., 2016; Lolla et al., 2021). Nevertheless, these few reports provide important insights into this novel post-translational modification and its physiological relevance in yeast and mammals. It is important to note, though, that the detection methods employed in those studies failed to confirm whether proteins could be in fact pyrophosphorylated *in vivo*. Notably, a novel phosphoproteomics approach has recently been reported, demonstrating its reliability in detecting pyrophosphorylated proteins isolated from human cell lines (Morgan et al., 2022). This breakthrough not only confirms the occurrence of protein pyrophosphorylation *in vivo*, but will also enable numerous applications in the field. Considerations on this method will be discussed in the subsequent section.

**Figure 2:**
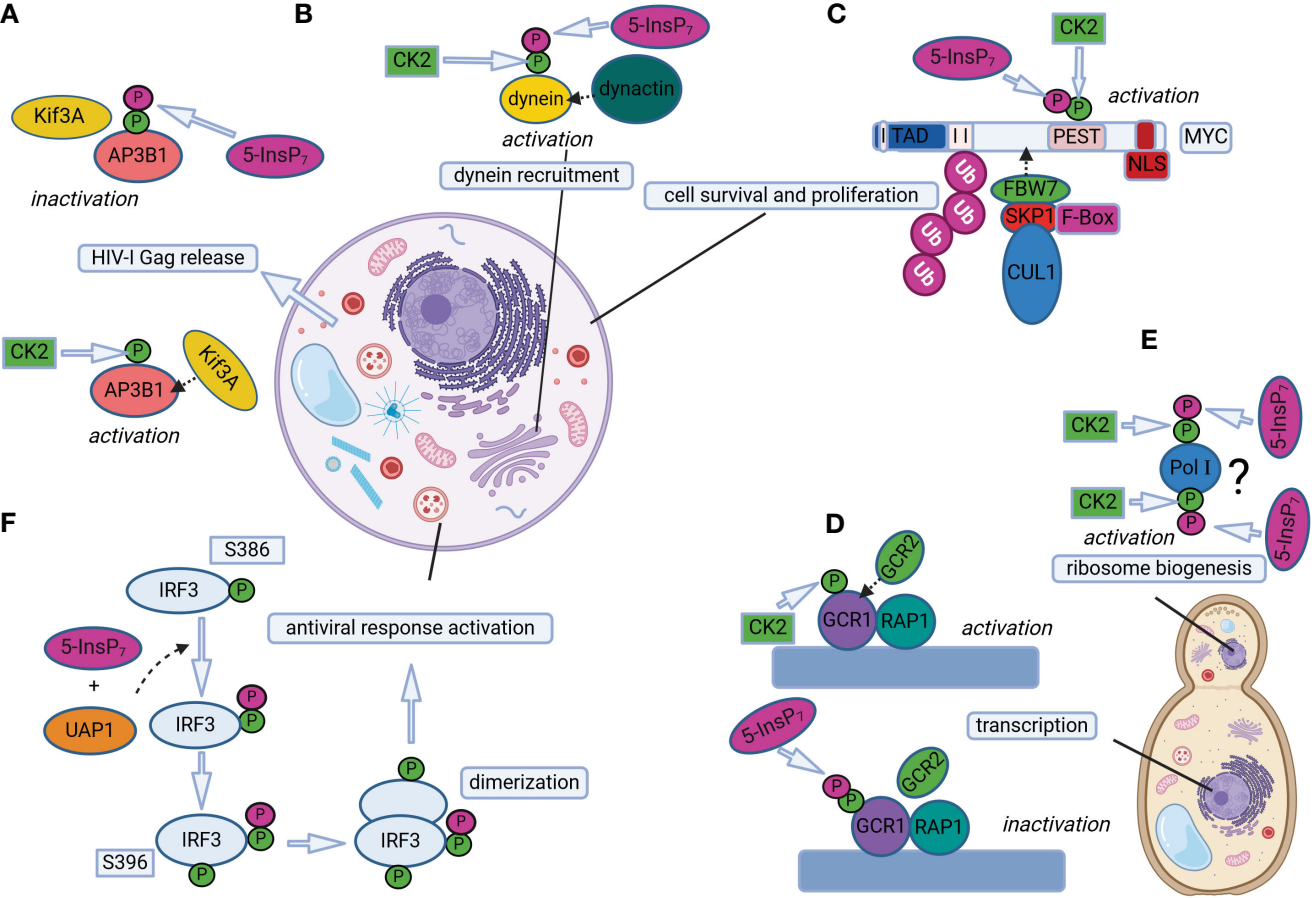
Protein pyrophosphorylation regulates different cellular processes in mammals and yeast. **(A)** 5-InsP_7_-mediated AP3B1 pyrophosphorylation regulates HIV-1 Gag release in human cells. Phosphorylation of AP3B1 by CK2 promotes AP3B1-Kif3A interaction, which in turn activates HIV-I Gag release out of the cell. Further pyrophosphorylation of CK2-primed AP3B1 by 5-InsP_7_ prevents AP3B1-Kif3A interaction, decreasing HIV-I Gag release. **(B)** 5-InsP_7_-mediated dynein pyrophosphorylation allows dynein-dynactin association in mammalian cells for regulating dynein-dependent cellular processes, such as endosomal sorting, Golgi morphology maintenance and vesicle trafficking. **(C)** Pyrophosphorylation of CK2-primed MYC promotes interaction of MYC and FBW7, a component of the SCF (Skp1/Cul1/F-box protein) E3 ubiquitin ligase complex. This interaction triggers MYC polyubiquitination and degradation, thereby regulating MYC levels to control cell survival and proliferation. **(D)** The binding of GCR1 and RAP1 to the promoter region of glycolytic genes is facilitated by GCR2 interaction with CK2 primed GCR1, which promotes transcription of glycolytic genes. On the other hand, InsP_7_-mediated pyrophosphorylation of GCR1 weakens GCR1/RAP1 association with GCR2, and transcription is repressed. **(E)** Putative phosphorylation of Pol I by CK2 could facilitate 5-InsP_7_-mediated pyrophosphorylation of Pol I, thereby regulating ribosome biogenesis. The question mark indicates that ribosome biogenesis could be controlled by InsP_7_-mediated pyrophosphorylation of Pol I at several sites and/or of unknown factors. **(F)** UAP1 catalyzes IRF3 pyrophosphorylation in the presence of 5-InsP_7_ to promote antiviral responses in mice cells. UAP1-mediated pyrophosphorylation of IRF3 at S386 promotes IRF3 phosphorylation at S396 and IRF3 dimerization. Pyrophosphorylation at S386 enhances UAP1 binding to TBK1, thereby promoting subsequent IRF3 phosphorylation at specific sites for inducing antiviral responses. The mammalian and fungal cell structures are reprinted from “generic animal cell and fungal cell”, by BioRender.com (2023). Retrieved from https://app.biorender.com/biorender-templates.

The first effort addressing the functions of InsP_7_-mediated pyrophosphorylation focused on the AP3B1 subunit of the human adaptor protein complex AP-3 (Azevedo et al., 2009). Adaptor proteins are involved in cargo sorting in vesicles, and the AP-3 complex in particular is specialized in localizing proteins to lysosomal compartments (Dell'Angelica et al., 1999). Results obtained from *in vitro* phosphorylation assays confirmed that AP3B1 is an InsP_7_ target primed via CK2-dependent phosphorylation at specific serine-rich acidic regions (**Figure 2A**), which were mapped at the subunit’s hinge domain (Azevedo et al., 2009). In an attempt to indirectly address *in vivo* pyrophosphorylation of AP3B1, “back-phosphorylation” experiments were performed. The rationale behind this method is the assumption that, should AP3B1 be endogenously pyrophosphorylated in InsP_7_-rich cells, the protein would be less prone to accept radiolabeled phosphate of 5β[^32^P]InsP_7_ *in vitro*, and vice-versa. To test this hypothesis, the AP3B1 subunit was expressed in *kcs1*Δ and *vip1*Δ yeast mutant strains, which lack or accumulate high levels of endogenous 5-InsP_7_ contents, respectively (Azevedo et al., 2009). The authors indeed observed a much stronger 5β[^32^P]InsP_7_-dependent pyrophosphorylation signal of AP3B1 derived from *kcs1*Δ extracts lacking 5-InsP_7_, when compared to WT-isolated AP3B1. On the other hand, *vip1*Δ mutants with high 5-InsP_7_ contents delivered AP3B1 that was barely phosphorylated by 5β[^32^P]InsP_7_ (Azevedo et al., 2009). An electrophoretic mobility shift was also observed in GST-AP3B1 expressed in WT and *kcs1*Δ yeast strains, which was interpreted as a further indication that AP3B1 is (pyro)phosphorylated by InsP_7_ *in vivo*. A gel shift of 5β[^32^P]InsP_7_-pyrophosphorylated AP3B1, however, was not visualized. This discrepancy was hypothesized to occur due to stoichiometry differences of the *in vitro* and *in vivo* experiments (Azevedo et al., 2009), or to uncharacterized components involved in this or possibly other post-translational modifications (Azevedo et al., 2009, 2015). To address the functional importance of InsP_7_-driven pyrophosphorylation of AP3B1, Kif3A, a motor protein from the kinesin superfamily was isolated as an AP3B1 interacting partner. The AP3B1-Kif3A association was then shown to be inhibited when AP3B1 was pyrophosphorylated, negatively affecting the Kif3A’s ability to drive the release of HIV1 particles from cells (Azevedo et al., 2009).

Dynein, which belongs to another class of motor proteins, has also been shown to be regulated by PP-InsPs in mammals and slime mold, thereby influencing vesicle transport (Chanduri et al., 2016). Cells with disrupted InsP_6_ kinase activity are defective in several dynein-dependent processes, such as cargo exit from early to recycling endosomes, Golgi morphology and vesicle mobility (Chanduri et al., 2016). The dynein intermediate chain 2C (IC-2C), a noncatalytic subunit which has been previously shown to be phosphorylated by CK2 (Karki et al., 1997; Vaughan et al., 2001), was then identified as a target of InsP_7_-dependent pyrophosphorylation at an N-terminal serine flanked by acidic residues (Chanduri et al., 2016). “Back-phosphorylation” assays from proteins isolated either from *Ip6k1* ^-/-^ or *Ip6k1* ^+/+^ mouse embryonic fibroblasts (MEFs, with low and normal PP-InsP contents, respectively), strongly suggest that IC-2C is also pyrophosphorylated *in vivo*. This modification was shown to promote IC-2C binding to the p150*^Glued^* subunit of dynactin (**Figure 2B**), a key dynein co-factor (Vaughan et al., 2001; Chanduri et al., 2016). In this case, weaker IC-p150*^Glued^* interactions were detected in *Ip6k1* ^-/-^ cells lacking PP-InsPs than in WT cells, and binding of dynein to membranes was decreased in the absence of PP-InsPs. Taken together, the results from Chanduri et al. (2016) indicate that 5-InsP_7_ positively modulates dynein-dynactin association and function via pyrophosphorylation of the dynein IC-2C subunit (**Figure 2B**).

Changes in protein-protein interactions by PP-InsP-dependent pyrophosphorylation were also reported to regulate transcription of glycolytic genes in budding yeast (Szijgyarto et al., 2011). However, in this, as well as in the previous work from the Saiardi’s lab (Azevedo et al., 2009), this post-translational modification destabilized the interaction of proteins to their binding partners (Szijgyarto et al., 2011). The mechanism proposed by the latter study involves the major glycolytic transcription factors GCR1, GCR2 and RAP1, the first of which can be pyrophosphorylated by InsP_7_ (**Figure 2D**). Both GCR1 and RAP1 bind to promoter regions of glycolytic genes, and this association is facilitated by interactions with GCR2, thereby promoting gene expression (**Figure 2D**). On the other hand, InsP_7_-driven pyrophosphorylation of GCR1 weakens the association of GCR1/RAP1 to GCR2, and transcription of glycolytic genes is repressed (Szijgyarto et al., 2011).

The identification of nucleolar proteins as targets of InsP_7_-dependent pyrophosphorylation (Saiardi et al., 2004; Bhandari et al., 2007) already spoke for a contribution of PP-InsPs in ribosome biogenesis, a process that starts in the nucleolus with transcription of pre-rRNA by RNA Polymerase I (Pol I) (Greber, 2016). Such an involvement is supported by the observation that yeast *kcs1*Δ mutants show disturbances in protein synthesis, most likely a result of the deranged ribosome biogenesis observed in these strains (Thota et al., 2015). This could be measured by the lower levels of rRNA transcription due to compromised elongation activity of Pol I and, consequently, reduced ribosomal content in *kcs1*Δ when compared to WT yeast. RNA Polymerase I was then identified as a target of InsP_7_-mediated pyrophosphorylation (**Figure 2E**), and putative CK2-primed serine residues were mapped on subunits A190, A43 and A34.5, mainly at C-terminal acidic clusters specific to Pol I (i.e., absent in Pol II, Pol III and respective subunit counterparts). Even though these sites were confirmed to accept the β-phosphate of 5β[^32^P]InsP_7_ in pyrophosphorylation assays, yeast mutant strains lacking the mapped pyrophosphoserines failed to reproduce the protein synthesis phenotype of *kcs1*Δ. This observation suggests that 5-InsP_7_ regulates Pol I-dependent transcriptional elongation via a combined effect, not only on the three subunits evaluated in the Thota et al. (2015) study, but potentially by pyrophosphorylating yet unidentified sites of other Pol I subunits (**Figure 2E**) and/or additional elongation factors (Thota et al., 2015). The molecular mechanisms by which transcriptional elongation of rRNA in yeast cells is influenced by InsP_7_-mediated pyrophosphorylation remain to be elucidated.

Another study from the Bhandari group reported that, in mammals, InsP_7_-dependent pyrophosphorylation also controls the levels of the MYC transcription factor by regulating its polyubiquitination preceding proteosomal degradation (Lolla et al., 2021). The InsP_7_-target site, termed by the authors as “pyro-phosphodegron”, was mapped to the C-terminus of the MYC’s PEST domain (**Figure 2C**), a region present in several short-lived proteins degraded via the ubiquitin pathway (Rechsteiner and Rogers, 1996; Meyer et al., 2011). In the case of MYC, pyrophosphorylation of the PEST domain by 5-InsP_7_ promotes its binding to FBW7, a component of the SCF (Skp1/Cul1/F-box protein) E3 ubiquitin ligase complex known to mediate the degradation of several proteins involved in cell fate (Koepp et al., 2001; Welcker and Clurman, 2007). The interaction of FBW7 to pyrophosphorylated MYC leads to MYC polyubiquitination and degradation, fine-tuning its levels to regulate cell survival and proliferation (Lolla et al., 2021). To exemplify this last conclusion, the increase of MYC stability via i) the expression of pyrophosphorylation-deficient MYC in *myc^-/-^* rat fibroblasts, as well as ii) by depleting InsP_7_ contents in *Ip6k1* ^-/-^ mice-derived spleens, led to increased cell death in conditions of cellular stress, and higher mitogen-stimulated cellular proliferation, respectively (Lolla et al., 2021).

Finally, pyrophosphorylation of the mice interferon regulatory factor 3 (IRF3) at one specific serine (Ser 386) was shown to be required for proper protein activation and function in promoting antiviral responses (Yang et al., 2023). Notably, IRF3 pyrophosphorylation is proposed to rely not only on 5-InsP_7_, but also on the activity of the metabolic enzyme UDP-N-acetylglucosamine pyrophosphorylase 1 (UAP1) (**Figure 2F**; Yang et al., 2023). These recent findings confront the notion of protein pyrophosphorylation being a non-enzymatic process, and indicate that unexplored additional factors could also promote PP-InsP-mediated protein pyrophosphorylation.

## 4 Recent advances in identifying protein pyrophosphorylation *in vivo*

In all studies addressing the functional relevance of InsP_7_-mediated pyrophosphorylation mentioned above, the actual *in vivo* occurrence of pyrophosphotransfer to proteins was implied from indirect measurements via “back-phosphorylation” assays. Despite being an elegant method that supports that endogenous InsP_7_ is involved in phosphotransfer reactions, ultimate conclusions taken by the usage of “back-phosphorylation” assays were accepted with caution by the scientific community. This skepticism can be justified by an alternative interpretation of the “back-phosphorylation” results, which takes in consideration evidences suggesting that CK2 activity can be modulated by inositol phosphates (Kim et al., 2006; Lee et al., 2008; Rao et al., 2014). As pointed out in a commentary by Stephen Shears (Shears, 2010), the high InsP_7_ levels in WT or *vip1Δ* yeast could have hindered CK2-mediated serine phosphorylation of AP3B1 *in vivo* (Azevedo et al., 2009), rendering a protein that is not primed to accept radiolabeled β-phosphate *in vitro*. On the other hand, in InsP_7_-depleted *kcs1*Δ strain, the unaffected CK2 activity would have led to monophosphorylated AP3B1 that could be robustly pyrophosphorylated *in vitro*. In other words, it might be possible that the “back-phosphorylation” assay did not reflect pyrophosphorylation of proteins, but a CK2-mediated monophosphorylation instead (Shears, 2010).

In the past decade, an impressive effort has been made to overcome the technical challenges pacing down the understanding of PP-InsP biology. The improvement of PP-InsP detection methods (Qiu et al., 2020, 2021; Riemer et al., 2021), as well as the development of new chemical tools, such as the synthesis of PP-InsPs analogs, standards and chiral selectors, have been crucial to uncover, e.g., PP-InsP enantiomeric identities, binding partners and function (Wu et al., 2013, 2014; Pavlovic et al., 2015; Brown et al., 2016; Pavlovic et al., 2016; Blüher et al., 2017; Harmel et al., 2019). Besides, the development of synthetic pyrophosphopeptides (Marmelstein et al., 2014) and affinity reagents for the enrichment of pyrophosphorylated proteins (Conway and Fiedler, 2015), combined with the optimization of mass spectrometry methods (Penkert et al., 2019) have been fundamental to the field, since these progresses finally enabled the *in vivo* detection of protein pyrophosphorylation (Morgan et al., 2022).

In the latter work, the Fiedler’s group established a novel workflow for pyrophosphoproteomics (Morgan et al., 2022) by systematically tackling the technical challenges of this approach. For instance, a set of synthetic standards was used to determine a characteristic neutral loss of pyrophosphopeptides, which was then used as a trigger for proteomic analyses (Penkert et al., 2017). This allowed, e.g., the distinction of peptides harboring one pyrophosphate moiety from peptides with two monophosphorylated residues, which was previously not possible due to their isobaric nature (Penkert et al., 2017). Besides, fragmentation parameters based on electron-transfer and higher energy collisional dissociation (EThcD) (Penkert et al., 2019), as well as sample preparation procedures were optimized to reliably identify pyrophosphosites in proteins derived from complex samples (Morgan et al., 2022).

The newly established pyrophosphoproteomics workflow was then employed with two human cell lines, i.e., HEK293T and HCT116, from which 108 and 78 pyrophosphosites distributed in 40 and 33 proteins were identified, respectively (Morgan et al., 2022). Subsequent sequence alignment and prediction analyses revealed that almost all pyrophosphoproteins harbor a CK2 consensus sequence. Some pyrophosphosites, however, are found in proline-directed kinase consensus sequences, and some were not predicted to be phosphorylated by any of those two types of kinases. These findings not only confirm the key role of CK2 in priming residues that undergo PP-InsP-dependent pyrophosphorylation, but also indicate that additional kinases could be involved in this process (Morgan et al., 2022). Another requisite for protein pyrophosphorylation that was recently proposed is that the target residues should lie within an intrinsically disordered region (IDR) of the protein (Ganguli et al., 2020). This hallmark could be confirmed by *in silico* analyses of protein targets identified in the pyrophosphoproteomics approach (Morgan et al., 2022), which predicted the localization of over 90% of the pyrophosphosites to IDRs. Finally, gene ontology analyses revealed that the great majority of the identified targets of protein pyrophosphorylation are involved in nuclear and nucleolar functions, including RNA processing and ribosome biogenesis (Morgan et al., 2022), which is in line with findings from previous studies, as mentioned above (Saiardi et al., 2004; Bhandari et al., 2007; Thota et al., 2015). In fact, two candidates were shown to be heavily pyrophosphorylated: the nucleolar and coiled-body phosphoprotein 1 (NOLC1), and the Treacher Collins syndrome protein 1 (TCOF1), both previously shown to undergo 5β[^32^P]InsP_7_-dependent pyrophosphorylation *in vitro* (Saiardi et al., 2004; Bhandari et al., 2007).

Is endogenous pyrophosphorylation of those proteins dependent on PP-InsPs? To assess this, three representatives (NOLC1, TCOF1 and the nuclear protein IWS1) were co-expressed with either active or inactive IPK6 in *Ip6k1* ^-/-^ HEK293T cells (i.e., lacking 5-InsP_7_), then purified and evaluated by neutral loss-triggered EThcD MS. As expected, protein fragments derived from cells expressing active IP6K presented several neutral-loss triggers that correspond to pyrophosphorylated residues in all three samples. In contrast, cells lacking 5-InsP_7_ failed to provide even a single peptide inferred to be pyrophosphorylated (Morgan et al., 2022). Besides, several pyrophosphorylated proteins, including NOLC1 and TCOF1, are annotated to localize to the nucleolar fibrillar center (FC), where InsP_6_ kinase isoforms also reside. Such a co-localization of IP6Ks and targets of protein pyrophosphorylation in the FC suggests that a local synthesis of 5-InsP_7_ would facilitate pyrophosphotransfer to proteins in this nucleolar sub-compartment.

The development of a technology that finally enables the detection of protein pyrophosphorylation by PP-InsPs *in vivo* opens new avenues for applications in different organisms, including plants. Nevertheless, the pyrophosphoproteomics approach is still evolving and some technical issues remain to be solved. For instance, as pointed out by Morgan et al. (2022), the level of candidate overlap between biological replicates can be improved. This already indicates that not all pyrophosphosites were identified, and that optimization of the method, e.g., by improving enrichment steps to exclude remaining non-pyrophosphorylated material from the samples is required.

## 5 Can plant proteins be pyrophosphorylated?

No evidence of PP-InsP-mediated pyrophosphorylation of plant proteins has been reported until now. Still, we find it appropriate to discuss a few aspects that suggest this novel post-translational modification might be relevant in plants as well. A key requirement for proteins to be pyrophosphorylated is the CK2-dependent priming of serine residues (Bhandari et al., 2007). The observation of this phenomenon in both yeast (Bhandari et al., 2007; Szijgyarto et al., 2011; Thota et al., 2015) and animal (Azevedo et al., 2009; Chanduri et al., 2016) systems, along with the structural conservation and wide eukaryotic distribution of CK2 protein kinases, could represent a starting point to explore the roles of protein pyrophosphorylation in plants. Protein kinase CK2, formerly called “casein kinase II”, is a serine/threonine kinase consisting of two catalytic subunits (*α* and/or *α’*) and two regulatory *β* subunits (Litchfield, 2003; Wang et al., 2022a). Differently than the few genes encoding the animal orthologs, the plant CK2 is a multigene family. For instance, the Arabidopsis *α* and *β* subunits of CK2 are each encoded by four genes (Salinas et al., 2006), rice CK2α and CK2β are encoded by four and two genes, respectively (Chen et al., 2015), and in maize, four isoforms encoding the CK2α subunit and three cDNA clones encoding the CK2β subunit have been identified (Riera et al., 2001; Łebska et al., 2009), conferring significant functional redundancy among the CK2 homologs (Salinas et al., 2006). CK2 phosphorylates proteins harbouring a minimal consensus sequence defined as Ser/Thr-X-X-Asp/Glu, where variations may also occur (Pinna, 2003). The substrate recognition is proposed to be mediated by a “basic cluster” that is unique to CK2 protein kinases, and is found in the catalytic subunits of mammalian and plant CK2 crystal structures (Niefind et al., 2001; Demulder et al., 2020).

Several CK2 targets have been identified in plants, the most of which are transcription factors or regulatory proteins (Mulekar and Huq, 2014; Wang et al., 2022a) involved in essential cellular processes such as protein translation and cell cycle regulation, as well as in development and environmental responses. Protein translation, for instance, was proposed to be regulated by CK2-dependent phosphorylation of several translation initiation factors, including eIF2α, eIF2β, eIF3c, eIF4B, eIF5, and the plant-specific histone deacetylase 2B (HD2B) in Arabidopsis and wheat (Browning et al., 1985; Dennis and Browning, 2009). Interestingly, the Morgan et al. (2022) study revealed an overrepresentation of this biological process in targets of InsP_7_-dependent pyrophosphorylation, including two translation initiation factors, namely EIF3C and EIF5B. Also pyrophosphorylated peptides from the mammalian Class I histone deacetylase 2 (HDAC2) were identified (Morgan et al., 2022). The latter does not share sequence homology to the Arabidopsis HD2B, but both proteins have been implicated in nuclear or nucleolar processes, which are in stark display among those modulated by pyrophosphorylated proteins (Saiardi et al., 2004; Bhandari et al., 2007; Thota et al., 2015). While mammalian HDAC2 is a key transcriptional regulator (Yamakawa et al., 2017), the plant HD2B has been shown to drive pre-RNA processing and ribosome biogenesis (Yamakawa et al., 2017; Chen et al., 2018). It is worth mentioning that structural and functional analyses of two additional members of the mammalian Class I histone deacetylases (i.e., HDAC1 and HDAC3) revealed that they are activated by inositol phosphates (Watson et al., 2012; Millard et al., 2013; Watson et al., 2016). In this case, InsPs bind allosterically to a pocket at the interface of HDACs and transcriptional co-repressor proteins. Also the budding yeast class I HDAC Rpd3L was shown to be activated by PP-InsPs (Worley et al., 2013), which are proposed to bind to a conserved InsP-binding site corresponding to those identified in crystal structures of the human homologs (Watson et al., 2012; Millard et al., 2013; Worley et al., 2013). No indication of PP-InsP-derived pyrophosphorylation was reported in those studies, though. Still, HDAC2 was unambiguously identified as a pyrophosphorylation target (Morgan et al., 2022), which could represent an additional layer of HDAC activity regulation. Moreover, a high-throughput phosphoproteomics approach in Arabidopsis and rice identified conserved phosphorylated peptides derived from AtHDA1/19 and Os06g38470, orthologs that belong to Class I HDACs (Nakagami et al., 2010). Intriguingly, several of those HDAC-derived peptides were predicted to be monophosphorylated and still displayed a negative charge of -4, instead of -2, which is expected for a single added phosphate (Nakagami et al., 2010). We are aware that such charge differences could derive from several factors. That being said, the hypothesis that they might be mirroring a pyrophosphate moiety is not unlikely. Besides, all the phosphoserines identified in the Arabidopsis and rice HDAC proteins lie within a highly acidic region (Nakagami et al., 2010) typically found in CK2-consensus sequences, and represent therefore potential targets of PP-InsP-derived pyrophosphorylation.

Important roles of CK2 in regulating inorganic phosphate (Pi) homeostasis have been reported in rice (Chen et al., 2015; Wang et al., 2020). Under sufficient Pi conditions, the rice CK2α3/β3 holoenzyme phosphorylates the PHOSPHATE TRANSPORTER OsPT8, thereby inhibiting its interaction with PHOSPHATE TRANSPORTER TRAFFIC FACILITATOR1 (OsPHF1). Such an interaction prevents OsPT8 exit from the ER to the plasma membrane, resulting in decreased Pi uptake (Chen et al., 2015). The authors also showed that the regulation of OsPT8 activity relies on the phosphorylation status of the CK2β3 subunit itself, via a negative feedback loop mechanism dependent on plant Pi status. That is, under Pi-limited conditions, unphosphorylated CK2β3 is degraded, which allows PT8-PHF1 interaction, facilitating OsPT8 exit to plasma membrane, ultimately leading to increase of Pi uptake (Chen et al., 2015). In this case, OsPT8 phosphorylation appears to require both regulatory and catalytic subunits of CK2α3/β3 (Chen et al., 2015). In a subsequent study, the rice OsCK2α3 subunit was shown to be sufficient to phosphorylate OsPHO2 (PHOSPHATE2), thereby facilitating its rapid degradation (Wang et al., 2020). OsPHO2 is homologous to the Arabidopsis ubiquitin-conjugating (UBC) E2 enzyme PHO2, which mediates degradation of high affinity phosphate transporters (Huang et al., 2013; Park et al., 2014), as well as of PHO1 (Liu et al., 2012), whose homologs are involved in Pi root-to-shoot translocation. The rice PHO1 protein was also identified as an OsPHO2 target and its degradation shown to regulate Pi homeostasis (Wang et al., 2020). Based on data resulting from both studies (Chen et al., 2015; Wang et al., 2020), the authors speculate that, under sufficient Pi conditions, CK2α3/β3 help maintain physiological Pi levels by promoting i) the retention of phosphorylated phosphate transporters (PTs) in the ER, thereby reducing Pi uptake from the soil, and with ii) Pi translocation for storage in vacuoles via OsPHO2-OsPHO1 pathway. In contrast, low internal Pi levels favor phosphorylation and de-stabilization of the CK2β3 subunit, which leads to a decrease in PT phosphorylation and subsequently its plasma membrane localization. In parallel, CK2β3 de-stabilization results in more “free” stand-alone CK2α3 that phosphorylates OsPHO2, further promoting Pi mobilization to shoots to sustain shoot growth (Wang et al., 2020). Several recent studies determined that inositol (pyro)phosphates play fundamental roles during phosphate starvation responses (PSRs) in several organisms, including plants. In Arabidopsis and rice, for instance, the promoter binding capacity of PHR transcription factors were proposed to be modulated by binding to SPX-domain containing proteins, a process that is dependent on PP-InsPs (i.e., by stabilizing the PHR-SPX interaction), therefore blocking expression of PSI genes at Pi-replete conditions (Wild et al., 2016; Dong et al., 2019; Zhu et al., 2019; Land et al., 2021; Riemer et al., 2021; Gulabani et al., 2022; Yang et al., 2024). Even though this transcriptional control occurs likely via PP-InsP allosteric binding to basic clusters at the surface of SPX domains (Wild et al., 2016), one cannot exclude that these molecules also modulate Pi responses at post-transcriptional levels downstream of PHR activation, in addition to SPX binding. Combining the knowledge that CK2 activity can be regulated by PP-InsPs in mammals (Rao et al., 2014) and that plant PP-InsP levels are drastically influenced by internal Pi conditions (Riemer et al., 2021), along with the roles CK2 play in Pi homeostasis, additional layers of PP-InsP-dependent regulation via, e.g., protein pyrophosphorylation could also be interesting to explore.

In light signalling, CK2 regulates plant development by phosphorylating positive regulators of photomorphogenesis, HY5 (ELONGATED HYPOCOTYL 5) and HFR1 (LONG HYPOCOTYL IN FAR-RED LIGHT 1) transcription factors in the presence of light (Hardtke et al., 2000) and by promoting degradation of the negative regulators, such as PIF1 (PHY INTERACTING FACTOR 1) (Bu et al., 2011). In line with this, phytochrome-B-mediated red-light signalling regulates plant phosphate acquisition by modulating HY5 transcription factors and phytochrome interacting factors (PIF) PIF4/PIF5 (Sakuraba et al., 2018). Future work will have to show whether PP-InsPs are involved in such a modulation and if CK2-dependent regulation of light signaling might also involve PP-InsP-dependent protein pyrophosphorylation.

A recent study reported that CK2 also phosphorylates MYC2, a master transcription factor involved in jasmonic acid (JA) signaling (Zhu et al., 2023). CK2-dependent phosphorylation triggered MYC2 binding to promoters of JA-responsive genes, regulating JA signaling and JA-dependent pathogen responses in Arabidopsis (Zhu et al., 2023). Interestingly, JA responses were reported to be also regulated by phosphorylation-coupled proteolysis of MYC2 (Zhai et al., 2013), a post-translational control that is common in mammalian systems but not fully explored in plants. It would be worth investigating whether also in plants, PP-InsPs are involved in this process, especially since InsP_7_-mediated pyrophosphorylation led to polyubiquitination and degradation of the mammalian MYC (Lolla et al., 2021). Besides, it is well established that PP-InsPs regulate JA-dependent responses in plants (Laha et al., 2015, 2016). In this case, InsP_8_, which is generated by the Arabidopsis PPIP5K VIH2, was proposed to be key to defenses against necrotrophic fungi and herbivore attack by acting as a co-ligand in the ASK1-COI1-JAZ1 receptor complex, whose binding to active JA-Ile during JA responses leads to de-repression of transcription factors, including MYC2 (Browse, 2009; Laha et al., 2015). This idea was corroborated by recent findings that *itpk1* mutants, which are defective in the synthesis of the InsP_8_ precursor 5-InsP_7_, and which display strong PSRs (Kuo et al., 2018; Riemer et al., 2021), display deranged JA-dependent responses as well (Pullagurla et al., 2023).

Finally, whether enzymes similar to the mammalian UAP1 (Yang et al., 2023) also regulate PP-InsP-dependent pyrophosphorylation of plant proteins is for now only an intriguing subject to be speculated. In Arabidopsis, deficiency in N-acetylglucosamine-1-P uridylyltransferases GlcNAc.UT1 and GlcNAc.UT2 enzymes (i.e., homologs of UAP1) result in defects in protein-N-glycosylation, sterility and hypersensitivity to salt (Chen et al., 2022). In rice, loss of UAP activity resulted in early leaf senescence and upregulation of defense-responsive genes, along with ROS accumulation and induction of JA and abscisic acid (ABA) biosynthesis (Wang et al., 2015, 2021). It remains to be shown whether these phenotypes are caused by deranged UDP-GlcNAc biosynthesis, and in consequence altered N-glycosylation, or whether protein pyrophosphorylation might also be involved.

## 6 Conclusions and perspectives

The hypothesis that proteins are subjected to a novel type of post-translational modification involving inositol pyrophosphates remained elusive for several years, as it was only recently that pyrophosphorylated proteins could be unambiguously identified from complex biological samples (Morgan et al., 2022). In that study, however, the mass spectrometry workflow was applied to mammalian cells, and also the initial efforts to indirectly measure protein pyrophosphorylation focused solely on yeast and metazoan proteins.

PP-InsPs have been implicated in a plethora of processes in plants, but the molecular mechanisms by which they perform their functions were only partially elucidated. In several cases, the mode of action utilized by these signalling molecules, i.e., whether they engage with proteins via allosteric binding, by outcompeting protein ligands, or via pyrophosphotransfer is unclear. In fact, there is so far no report on pyrophosphorylated plant proteins. This made us wonder how to approach this topic, and we pondered that a good starting point would be looking at the protein kinase CK2. We consider the broad distribution of CK2 homologs in plants, as well as the common pathways regulated by CK2 activity and PP-InsP signalling, hints that PP-InsP-mediated pyrophosphorylation also takes place in plants. It is still an open question whether PP-InsPs are involved in the regulation of plant CK2 itself or, importantly, if the CK2 protein targets are prone to further pyrophosphorylation. It would also be worth investigating whether plant PP-InsP-generating enzymes co-localize with CK2 subunits, as well as with putative targets of pyrophosphorylation. In the work of Rao et al. (2014), phosphorylation of members of the TTT (Tel2, Tti1, Tti2) cochaperone family by CK2 is regulated by selective binding of the protein kinase to IP6K2-derived 5-InsP_7_. IP6K2 was shown to directly associate with Tti1 but no physical interaction between CK2 and IP6K2 has been reported in this case. Nevertheless, these results strongly support that IP6K2, CK2 and its targets should reside in close proximity in order to promote downstream responses (Rao et al., 2014). Moreover, a recent protein interactome study revealed that the human IP6K1 interacts with both catalytic and regulatory CK2 subunits, as well as with several known pyrophosphorylation substrates, such as AP3B1, NOLC1, TCOF1 (Hamid et al., 2023). These findings provide important clues on the regulation of protein pyrophosphorylation, via a process that relies on localized synthesis of 5-InsP_7_ concomitant with a CK2-dependent priming of serine residues (Hamid et al., 2023). While the Arabidopsis InsP_6_ kinase ITPK1 physically interacts with the TIR1 F-box protein component of the auxin receptor complex (Laha et al., 2022), it remains unclear whether this interaction evolved to stimulate auxin signaling by local production of 5-InsP_7_ as a co-ligand of the auxin receptor or whether locally produced 5-InsP_7_ also pyrophosphorylates phosphoserine residues in this complex (Laha et al., 2022). Further, we believe that, despite a full repertoire of PP-InsPs including a 4/6-InsP_7_ not present in yeast and just recently identified in mammals (Riemer et al., 2021; Danye et al., 2022), as well a new PP-InsP4 isomer of unknown isomer identity (Riemer et al., 2021), the occurrence of this post-translational modification in plants has likely been masked by methodological challenges that hindered the detection of pyrophosphorylated proteins. It remains an interesting question whether different PP-InsP isomers have different substrate specificities towards their phosphoserine-containing protein targets. The seminal work of Bhandari et al. (2007) suggests no major differences in protein phosphorylation patterns when protein extracts obtained from budding yeast were incubated with either radiolabeled 5-InsP_7_ or Vip1-dependent InsP_8_ or 1-InsP_7_ (initially wrongly annotated as 4/6-InsP_7_), suggesting no differences in substrate specificities. However, the autoradiography of gel-separated protein extracts employed at the time was not very refined and reported only strong phosphorylation events, but not less abundant or weaker targets of PP-InsP-dependent pyrophosphorylation. In addition, 4/6-InsP_7_, or different PP-InsP_4_ isomers were not employed in these experiments. We envision that by taking advantage of recent advances in pyrophosphoproteomics, and by conducting similar *in vitro* experiments but also by analyzing plants with contrasting PP-InsP levels, could provide valuable insights for exploring this exciting role of PP-InsPs in plants. At last, and maybe most importantly, it will be an exciting endeavour to not only identify and map pyrophosphorylation sites in plants, but to learn more about the consequences of this modification on protein function and/or localization, and how this might influence cellular metabolism, growth and development, as well as responses to external stimuli, and in consequence, plant phenotypes in different environments.

## Author contributions

YM: Writing – original draft, Writing – review & editing. GS: Funding acquisition, Writing – original draft, Writing – review & editing. MK: Conceptualization, Writing – original draft, Writing – review & editing.

## Glossary

**Table:**

CDP-DAG	CDP-diacylglycerol
DIPP	diphosphoinositol polyphosphate phosphohydrolase
DSP	dual-specificity phosphatase
EThcD	electron-transfer and higher energy collisional dissociation
GlcNAc.UT	N-acetylglucosamine-1-P uridylyltransferase
Glu(6)P	glucose-6-phosphate
HDAC	histone deacetylase
IMP	inositol monophosphatase
InsPs	inositol phosphates
IP6K	inositol hexakisphosphate kinase
IPK2	inositol polyphosphate 6/3-kinase
IP_3_-3K	inositol 1,4,5-trisphosphate 3-kinases
ITPK	inositol (1,3,4) trisphosphate 5/6-kinase
MEFs	mouse embryonic fibroblasts
MIPS	*myo*-inositol phosphate synthase
PFA-DSPs	plant and fungi atypical dual-specificity phosphatases
PIS	phosphatidylinositol synthase
PLC	phospholipase C
Pol I	RNA polymerase I
PP-InsPs	inositol pyrophosphates
PPIP5K	diphosphoinositol-pentakisphosphate kinase
PSI	Pi starvation-induced
PSR	phosphate starvation response
PT	phosphate transporter
PtdIns	phosphatidylinositol
UAP	UDP-N-acetylglucosamine pyrophosphorylase
